# Acute Percutaneous Repair of Medial Collateral Ligament With Suture Augmentation in the Multiligamentous Injured Knee Results in Good Stability and Low Rates of Postoperative Stiffness

**DOI:** 10.1016/j.asmr.2023.100799

**Published:** 2023-10-04

**Authors:** Kurt Holuba, Sebastian Rilk, Harmen D. Vermeijden, Robert O’Brien, Jelle P. van der List, Gregory S. DiFelice

**Affiliations:** a Department of Orthopaedic Surgery, Hospital for Special Surgery, New York, New York, U.S.A.; b Medical University of Vienna, Vienna, Austria; c Department of Orthopaedic Surgery, Amsterdam UMC location University of Amsterdam, Amsterdam, the Netherlands; d Amsterdam Movement Sciences, Sports, Amsterdam, the Netherlands

## Abstract

**Purpose:**

To assess the clinical and patient-reported outcome measures (PROMs) of acute superficial medial collateral ligament (sMCL) repair with suture augmentation (SA) in the setting of a multiligamentous injured knees (MLIKs) at 2-year follow-up.

**Methods:**

A retrospective analysis of consecutive patients with MLIK with grade III sMCL injuries who underwent acute (<6 weeks) sMCL repair with SA was conducted. Clinical follow-up was performed at minimum 1-year postoperatively, and PROMs were collected at the latest follow-up (minimum 2 years’ postoperatively). Continuous variables were reported in median with interquartile range (IQR).

**Results:**

A total of 20 patients (41.4 [28.5-47.9] years of age) with grade III sMCL injury and additional injury to 1 cruciate ligament (KDI-M; n = 13) or bicruciate (KDIII-M; n = 7) were enrolled with a median follow-up of 4.3 (3.6-5.2) years. In total, 90% (n = 18) of patients with MLIK treated with acute sMCL repair and early range of motion rehabilitation protocol demonstrated negative valgus laxity stress testing in 0 and 30° flexion and low reoperation rates (n = 1, 5%) due to stiffness. In addition, good-to-excellent subjective outcomes were reported at final follow-up: median International Knee Documentation Committee 82.2 (78.7-90.8), Lysholm 95.0 (90.0-100.0), modified Cincinnati Score 89.0 (83.3-96.0), Single Assessment Numeric Evaluation 90.0 (83.8-95.0), Forgotten Joint Score 79.2 (62.5-91.7), Tegner 5.0 (IQR 4.0-6.0), and ACL-Return to Sport after Injury Scale 78.3 (IQR 66.7-90.0).

**Conclusions:**

In this study, 20 heterogenous patients with MLIKs treated with acute percutaneous sMCL repair with SA had excellent stability, low rates of postoperative stiffness, and good-to-excellent PROMs at short-term follow-up.

**Level of Evidence:**

Level IV, therapeutic case series.

The medial collateral ligament (MCL) is one of the most frequently injured ligaments in the knee joint and is seen in more than 40% of all multiligamentous injured knees (MLIKs).^1^ The MCL is the knee’s primary restraint against valgus motions and is crucial to the distribution of mechanical stress across the knee joint.^2^, ^3^, ^4^, ^5^, ^6^ Nonoperative treatment for isolated partial (grade I and II) superficial medial collateral ligament (sMCL) tears is widely agreed upon, given the intrinsic healing capacity of the ligament.^7^ However, the treatment paradigm of severe (grade III) sMCL and/or deep medial collateral ligament (dMCL) injuries is still under debate, especially in the MLIK setting.^8^, ^9^, ^10^, ^11^, ^12^ For these high-grade MLIKs, it remains controversial whether to treat the sMCL nonoperatively or operatively. Historically, outcomes of acute MCL repair led to high rates of postoperative stiffness.^13^, ^14^, ^15^, ^16^, ^17^ As a result, the currently accepted treatment algorithm is nonoperative treatment of the MCL followed by delayed reconstruction of concomitant ligamentous injuries.^12^^,^^14^^,^^18^, ^19^, ^20^

However, this approach results in a longer time to surgery and therefore an increased risk for residual valgus laxity.^18^^,^^21^, ^22^, ^23^ Several biomechanical and clinical studies have further shown that residual medial-sided laxity places additional stress on the cruciate ligaments, leading to an increased risk of graft failure.^2^, ^3^, ^4^, ^5^, ^6^ To address these issues, the concept of acute sMCL repair has been introduced.^18^^,^^24^^,^^25^ With recent advancements in surgical techniques and rehabilitation protocols, improved clinical outcomes are to be expected.^18^ In addition, implementation of suture augmentation (SA) devices provide early knee stability to facilitate anatomic healing of the sMCL and allow for early and safe range of motion (ROM).^25^, ^26^, ^27^, ^28^, ^29^, ^30^ Although promising, a lack of studies addressing acute percutaneous sMCL repair with SA exist.

The purpose of this study was to assess the clinical and patient-reported outcome measures (PROMs) of acute sMCL repair with SA in the setting of a MLIK at 2-year follow-up. We hypothesized that patients treated with acute sMCL repair with SA would have minimal valgus laxity and low rates of postoperative stiffness.

## Methods

### Patient Selection

Institutional review board approval was obtained for this retrospective study (#2017-0404), and informed consent was obtained from each patient enrolled. Patients with MLIK injuries who underwent acute percutaneous sMCL repair with SA, between December 2008 and April 2021 at a single center by the senior author (G.S.D.), with a time from injury to surgery of <6 weeks, were considered for eligibility. Exclusion criteria applied were sMCL reconstruction instead of repair due to insufficient sMCL tissue quality; staged surgery procedure; delayed (>6 weeks) surgery; or insufficiently obtained follow-up (clinical follow-up <1 year and/or PROM evaluation <2 years postoperatively). The indications for acute percutaneous sMCL repair with SA were as previously reported: clinical confirmation of MCL tear and magnetic resonance imaging suggesting grade III injury.^24^^,^^25^ Concomitant injuries to the anterior cruciate ligament (ACL), posterior cruciate ligament (PCL), and/or lateral collateral ligament were included and described according the Schenck Classification.^31^ All data were collected in a prospectively managed database of the senior author (G.S.D.), including preoperative clinical and demographic characteristics, as well as intraoperative information.

### Surgical Technique

All surgeries were performed by the senior author (G.S.D.), a fellowship-trained sports traumatology surgeon with 20+ years of experience. Primary repair of the sMCL with SA was performed in patients with complete (grade III) ruptures at either the femoral or tibial insertion points. This technique has been previously described in detail.^24^^,^^25^ For midsubstance, complex grade III ruptures, or sMCL tears with insufficient tissue quality, MCL reconstruction was performed. For proximal or distal avulsions, 2 small percutaneous incisions were made over the femoral and tibial sMCL insertion sites. Depending on the avulsion site, repair stitches were passed in a Bunnell-type pattern toward the avulsion site. The repair sutures were then passed through the eyelet of a 4.75-mm Vented BioComposite SwiveLock suture anchor (Arthrex, Naples, FL), which was loaded with FiberTape (Arthrex). The sMCL was then tensioned and the suture anchor was deployed. Finally, the FiberTape was passed to the opposite insertion site under layer 1 and running superficial to the sMCL, and secured using a second suture anchor. As for mixed ruptures, repair stitches were placed along the entire length of the damaged tissue, toward either the proximal or distal insertion sites, depending on the nearest site to the injured tissue. The sutures were secured using a preloaded suture anchor, as described previously. For concomitant medial-sided injuries of the medial patellofemoral ligament, posterior oblique ligament (POL), or dMCL, a similar repair technique was used. The dMCL and/or POL was sutured directly towards the sMCL avulsion site, where the repair stitches were loaded into the initial suture anchor used to secure the sMCL. The remaining core sutures were then be used to repair the medial patellofemoral ligament, if necessary.

As for the ACL and PCL, a standard treatment algorithm was used depending on tear location.^32^ For proximal or distal avulsion tears with sufficient tissue length and quality to be reapproximated back to the femoral or tibial footprint, primary repair with or without SA was performed, as previously described.^33^^,^^34^ All other tear types underwent standard ACL or PCL reconstruction using anterior tibialis or semitendinosus allograft tendons. No patients with lateral collateral ligament or posterolateral corner injuries were included.

### Postoperative Rehabilitation

All patients followed a standardized milestone-based rehabilitation protocol, with slight modifications depending on injury pattern. Postoperatively, all patients wore a hinged brace, which was locked in extension during ambulation. Gentle ROM and quadriceps strengthening exercises started directly after surgery. When protective quadriceps strength returned, the brace was unlocked and weight-bearing was gradually increased. Full weight-bearing was allowed depending on the patient’s progress and injury pattern but generally occurred between 4 and 8 weeks. Physical therapy was then intensified to improve ROM and muscle strength. Patients were regularly assessed during clinical visits by the senior author (G.S.D.) at 1 week, as well as at 1, 3, 6, 9, and 12-months’ postoperatively, where ROM, quadriceps muscle strength, Pivot shift, posterior drawer, varus/valgus stress testing (0 and 30°), and functional milestones were used as basis for progression. Generally, patients were ready to return to play at roughly 9 to 12 months after surgery.

### Clinical Evaluation and Data Collection

Each patient was contacted and invited for clinical evaluation of knee laxity and collection of PROMs, beginning at 1-year postoperatively. The senior author (G.S.D.) performed all clinical evaluations. PROMs consisted of the International Knee Documentation Committee Subjective Score,^35^ Lysholm score,^36^ modified Cincinnati Score,^37^ Single Assessment Numeric Evaluation,^38^ Forgotten Joint Score,^39^ preinjury and current Tegner Activity Scales,^40^ and the ACL-Return to Sport After Injury Scale.^41^ In a case in which a 1-year clinical examination had been completed but a 2-year clinical examination could not be scheduled, a standardized telemedicine interview was conducted (between July 2021 and April 2023). During these interviews, PROMs were taken and patients were asked if they had sustained any type of reinjury or complication, underwent subsequent surgeries, or experienced recurrent subjective instability. If any of these were true, the patient was scheduled for an in-office visit to undergo physical examination. In addition, the medical records of all patients were reviewed to record demographics, injury patterns, and surgical details.

### Statistical Analysis

Statistical analyses were performed using SPSS Statistics, Version 28 (IBM Corp., Armonk, NY). All continuous variables were described with median and interquartile range (IQR), and nominal variables were described with frequencies (%).

## Results

### Patient Demographics

**Fig 1:**
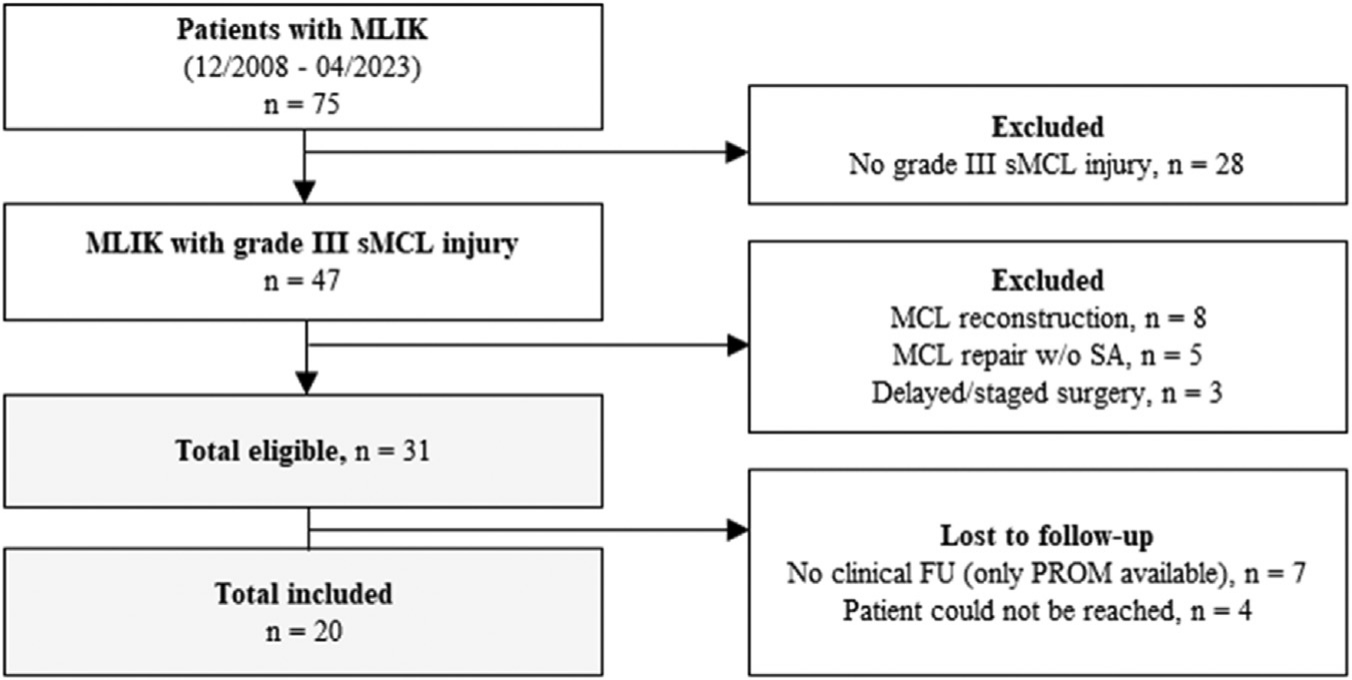
Flow diagram for inclusion and exclusion of patients. (FU, follow-up; MCL, medial collateral ligament; MLIK, multiligamentous injured knee; PROM, patient-reported outcome measure; sMCL, superficial medial collateral ligament.)

Between December 2008 and April 2021, 75 patients underwent MLIK surgery, of whom 47 were diagnosed with additional sMCL injury (Fig 1). Eight patients were referred for MCL reconstruction, 5 for repair without SA and 3 for delayed or staged surgery and therefore not considered for study inclusion. Thirty-one patients met final inclusion criteria, of whom 11 were lost to follow-up. A total of 20 (65%) consecutive patients (41.4 [28.5-47.9] years of age), with a median time to surgery of 10.5 (9.0-16.5) days and a median follow-up of 4.3 (3.6-5.2) years were included in the final data analysis. Thirteen patients (65.0%) had sustained a single cruciate ligament with an additional MCL injury (KDI-M) (12 ACL, 1 PCL), and 7 patients (35.0%) had bicruciate injuries with an additional MCL injury (KDIII-M). Concerning the tear location of sMCL injuries, femoral avulsions were most common (75.0%), followed by distal avulsions (15.0%), and mixed tear patterns (10.0%), which consisted of combined distal and proximal tears. Of these, 5 patients (25.0%) had solely repair of the sMCL, 3 (15.0%) sMCL and dMCL repair, 8 (40.0%) sMCL, dMCL, and POL repair, and 4 (20.0%) required sMCL and POL repair. An extensive overview of demographic and baseline characteristics is outlined in Table 1.

**Table 1:** Patient Demographics and Injury Patterns

Pt.	Demographics					Preoperative Characteristics			Operative Procedures						
	Sex	Age at Surgery, y	BMI	Injury to Surgery, d	Latest FU, y	MCL Tear Location	Preop. Valgus Laxity, 0°/30°	Schenck Classification	sMCL	dMCL	POL	ACL	PCL	Menisci	Additional
1	M	51.4	27.9	26	7.0	Proximal	2+/2+	KD3-M	Repair w/ SA			ACLR	PCLR		
2	F	36.4	24.1	9	6.0	Proximal	3+/3+	KD3-M	Repair w/ SA	Repair	Repair	ACLPR-SA	PCLPR		MPFL repair; MFL repair
3	M	66.2	29.1	9	5.4	Proximal	3+/3+	KD3-M	Repair w/ SA	Repair	Repair	ACLPR-SA	Conservatively	B/L PM	CP
4	M	51.6	21.5	9	4.8	Proximal	3+/3+	KD1-M	Repair w/ SA	Repair	Repair	ACLR		Lat. PM	CP
5	F	42.4	27.0	14	4.7	Proximal	3+/3+	KD1-M	Repair w/ SA		Repair	ACLPR-SA		Lat. PM	
6	F	41.1	19.8	11	4.2	Proximal	3+/3+	KD1-M	Repair w/ SA		Repair	ACLR			CP
7	F	19.2	22.1	20	4.5	Proximal	3+/3+	KD3-M	Repair w/ SA	Repair		ACLPR-SA	PCLPR-SA	Lat. repair	MPFL repair
8	F	29.1	19.5	9	4.0	Proximal	3+/3+	KD1-M	Repair w/ SA	Repair		ACLR			
9	F	41.8	21.6	8	3.6	Proximal	3+/3+	KD1-M	Repair w/ SA		Repair	ACLPR-SA		Lat. repair	
10	F	46.7	25.0	10	3.5	Proximal	3+/3+	KD3-M	Repair w/ SA	Repair	Repair	ACLPR-SA	PCLPR	Lat. repair	MPFL repair
11	F	43.0	22.0	10	3.3	Proximal	3+/3+	KD1-M	Repair w/ SA	Repair	Repair	ACLPR-SA		Lat. PM	
12	M	31.4	28.1	18	2.8	Proximal	2-3+/3+	KD1-M	Repair w/ SA	Repair	Repair	ACLPR-SA		Lat. PM	
13	F	43.9	26.6	25	4.2	Proximal	3+/3+	KD1-M	Repair w/ SA			ACLR		Lat. PM	CP
14	F	23.5	20.6	9	6.0	Proximal	2+/2-3+	KD1-M	Repair w/ SA			ACLPR-SA			
15	F	27.0	27.3	5	5.1	Proximal	3+/3+	KD1-M	Repair w/ SA		Repair	ACLR			MPFL repair
16	F	24.7	33.4	16	4.0	Distal	3+/3+	KD3-M	Repair w/ SA			ACLPR-SA	PCLPR-SA	Med. Repair	
17	M	52.0	47.2	16	3.1	Distal	3+/3+	KD1-M	Repair w/ SA	Repair		ACLPR-SA		Med. PM	CP
18	M	19.9	20.7	20	4.5	Distal	3+/3+	KD3-M	Repair w/ SA	Repair	Repair	ACLR	PCLPR		
19	M	69.5	22.4	7	6.0	Mixed	3+/3+	KD1-M	Repair w/ SA				PCLPR	B/L PM	Removal of loose bodies
20	F	33.1	20.2	15	1.8	Mixed	3+/3+	KD1-M	Repair w/ SA	Repair	Repair	ACLPR-SA		B/L Repair	MPFL repair; CP
Median		41.4	23.2	10.5	4.3										
IQR		28.5-47.9	21.3-27.4	9-16.5	3.6-5.2										

ACLPR, anterior cruciate ligament primary repair; ACLR, anterior cruciate ligament reconstruction; B/L, bilateral; CP, chondroplasty; dMCL, deep medial collateral ligament; F, female; FU, follow-up; Lat., lateral; M, male; MCL, medial collateral ligament; MCLR, medial collateral ligament repair; Med., medial; MPFL, medial patellofemoral ligament; PCLPR, posterior collateral ligament primary repair; PCLR, posterior collateral ligament reconstruction; PM, partial meniscectomy; Pt., patient; SA, suture augmentation; sMCL, superficial medial collateral ligament.

### Clinical Outcomes

Clinical outcomes are outlined in Table 2. At latest clinical follow-up (4.2 [3.0–4.9] years) valgus laxity stress test in 0° and 30° flexion was stable in 18 patients (90.0%), grade I in 2 (10.0%), and grade II or grade III (= clinical failure) in zero patients (0.0%). ROM testing revealed no patients with significant extension or flexion deficits, yet 1 patient (5.0%) required manipulation under anesthesia and lysis of adhesions. In addition, 1 patient (5.0%) underwent subsequent unicondylar knee arthroplasty at 2.4 years after the initial MLIK procedure.

**Table 2:** Clinical Outcomes

Pt.	FU, y	ROM, °	Lachman	Lachmeter, mm	Valgus, 0°/30°	PDT	IL ACL Failure	CL ACL Failure	MCL Failure	Reoperation/Complication
1	5.6	0-135	Neg		0/1+∗	1+				
2	6.0	0-140	Neg		Stable	3+				
3	5.4	0-110	Neg		Stable	Neg				Medial UKA
4	4.8	0-140	Neg	0.94	Stable	Neg				
5	4.7	0-140	Neg	1.28	Stable	Neg				
6	4.2	0-135	Neg	0.85	1+/1+	Neg				
7	4.5	0-140	†		Stable	Neg	X			
8	4.0	0-150	Neg	0.03	Stable	Neg				
9	3.6	0-150	Neg	0.94	Stable	Neg				
10	1.0	0-140	Neg		Stable	1+				
11	1.3	2-135	Neg	2.19	Stable	Neg				
12	2.8	0-145	Neg	0.52	Stable	Neg				
13	4.2	0-135	1+	1.48	Stable	Neg				
14	6.0	0-145	†		Stable	Neg	X			
15	1.0	0-140	Neg		Stable	Neg				
16	4.0	0-140	Neg	-2.15	Stable	Neg				
17	3.1	0-130	Neg	0.21	Stable	Neg				
18	4.5	0-140	†		Stable	Neg	X			
19	6.0	0-140	Neg		Stable	Neg				MUA + LOA
20	1.8	0-145	†		Stable	Neg	X			
Median	4.2			0.90						
IQR	3.0-4.9			0.28-1.2						

ACL, anterior cruciate ligament; bilateral; CL, contralateral; FU, follow-up; IL, ipsilateral; LOA, lysis of adhesions; MUA, manipulation under anesthesia; MCL, medial collateral ligament; PDT, posterior drawer test; Pt., patient; ROM, range of motion; UKA, unicondylar knee arthroplasty.

∗ Symmetric to contralateral knee.

† Patient underwent revision ACL reconstruction.

Outcomes for cruciate ligaments revealed that 4 patients (21.1%) treated for ACL injury had failed at a mean of 3.6 years after surgery, 3 of whom had undergone ACL primary repair with SA and 1 allograft ACL reconstruction. All 4 patients underwent revision ACL reconstruction. Considering PCL survival, 1 patient (12.5%) presented with subsequent injury, which was treated conservatively.

### Patient-Reported Outcome Measurements

PROMs were excellent, at a median final follow-up of 4.2 (3.6-5.2) years, with median International Knee Documentation Committee 82.2 (78.7-90.8), Lysholm 95.0 (90.0-100.0), modified Cincinnati Score 89.0 (83.3-96.0), Single Assessment Numeric Evaluation 90.0 (83.8-95.0), Forgotten Joint Score 79.2 (62.5-91.7), Tegner 5.0 (IQR 4.0-6.0), and ACL-Return to Sport after Injury Scale 78.3 (IQR 66.7-90.0). PROMs for each patient are detailed in Table 3.

**Table 3:** PROM Outcomes

Pt.	FU, y	IKDC	Lysholm	Cincinnati	SANE	FJS	Tegner Pre-	Tegner Post-	ACL-RSI
1	7.0	77.0	99	86	80	79.2	5	5	66.7
2	6.0	73.6	90	71	80	56.3	7	4	
3	5.4	65.5	85	80	85	75.0	4	4	
4	4.8	85.1	95	94	100	95.8	7	6	68.3
5	4.7	97.7	90	100	90	91.7	6	6	81.7
6	4.2	79.3	100	89	80	81.8	6	5	10.0
7∗									
8	4.0	88.5	95	94	90	79.2	3	3	63.3
9	3.6	94.3	98	100	90	80.0	6	5	97.9
10	3.5	80.5	95	89	99	N/A	6	6	93.8
11	3.3	79.3	95	81	95	36.7	5	4	81.3
12	2.8	100.0	100	100	95	91.7	10	10	100.0
13	4.2	82.8	100	86	90	56.3	6	6	41.7
14∗									
15	5.1	81.6	74	80	85	33.3	3	3	
16	4.0	90.8	100	96	85	97.9	7	6	90.0
17	3.1	90.8	100	96	98	95.0	6	5	77.1
18∗									
19	6.0	66.7	83	84	80	68.8	6	6	78.3
20∗									
Median	4.2	82.2	95.0	89.0	90.0	79.2	6.0	5.0	78.3
IQR	3.6-5.2	79.7-90.8	90.0-100.0	83.3-96.0	83.8-95.0	62.5-91.7	5.0-6.3	4.0-6.0	66.7-90.0

ACL, anterior cruciate ligament; ACL-RSI, ACL-Return to Sport after Injury Scale; Cincinnati, Modified Cincinnati Rating System; FJS, Forgotten Joint Score; FU, follow-up; IKDC, International Knee Documentation Committee; IQR, interquartile range; Lysholm, Lysholm Score; PROM, patient-reported outcome measure; Pt., patient; SANE, Single Assessment Numeric Evaluation; Tegner, Tegner Activity Scale.

∗ Patient underwent revision ACL reconstruction.

## Discussion

The most important finding of this study was that 90% (n = 18) of patients with MLIK treated with acute sMCL repair with SA and early ROM rehabilitation protocol demonstrated negative valgus laxity stress testing in 0° and 30° flexion and low reoperation rates (n = 1, 5%) due to stiffness. In addition, good-to-excellent subjective outcomes were reported at final follow-up (median 4.3 [3.6-5.2] years).

Detractors of acute MCL repair cite historically high rates of stiffness as the main concern to this approach.^13^, ^14^, ^15^, ^16^, ^17^ In this study, acute percutaneous sMCL repair with SA provided valgus stability with a lower rate of stiffness (5.0%) than reported historically. Comparing these results with modern clinical outcomes is limited due to heterogeneity of MCL repair techniques. Most modern techniques do not use SA technology and are more invasive, achieving repair through an incision over the entire length of the MCL.^42^, ^43^, ^44^, ^45^, ^46^, ^47^, ^48^, ^49^, ^50^, ^51^, ^52^, ^53^ Furthermore, these techniques vary greatly in their time to surgery, indicated patient populations, reported outcomes, and rehabilitation protocol. Nonetheless, outcomes of MCL repair are homogenized in the literature. A recent meta-analysis compared outcomes of MCL repair, MCL reconstruction, or nonoperative treatment in MLIKs.^9^ Of the 8 studies labeled as MCL repair, 4 studies described a curvilinear medial incision over the length of the MCL,^44^, ^45^, ^46^, ^47^ 3 studies did not include a full explanation of surgical technique,^6^^,^^22^^,^^54^ and 1 study used a small incision over the femoral sMCL attachment to achieve repair, albeit without the additional stability provided by suture anchors and SA.^55^ Similarly, a recent systematic review described outcomes of surgical MCL repair, regardless of time to surgery or concomitant ligament damage. Of the 355 knees evaluated across 16 studies, fixation was achieved by suture-only repair in 49.5% (n = 176, 6 studies), staples in 12.1% (n = 43, 2 studies), suture anchors in 11.2% (n = 40, 2 studies), and mixed or unknown fixation in 27% (n = 96, 6 studies).^56^ Given the heterogeneity of modern MCL repair techniques, stiffness can be seen in 0% to 27% of patients, and MCL failure in 0% to 35%, which continues to complicate the decision as whether the MCL should be treated operatively or nonoperatively.^42^, ^43^, ^44^, ^45^, ^46^, ^47^, ^48^, ^49^, ^50^, ^51^, ^52^, ^53^ Comparable outcomes for the MCL technique used in the current study is limited, as only biomechanical analysis and a single case report exists.^28^^,^^29^^,^^57^

In the current study, acute sMCL repair with SA resulted in negative valgus laxity in 90% (n = 18) of patients. This outcome bolsters the argument against nonoperative MCL treatment, as residual valgus laxity can be seen when the MCL is treated nonoperatively.^18^^,^^21^, ^22^, ^23^ Halinen et al.^22^ compared nonoperative treatment versus acute MCL repair in 47 patients with ACL-MCL injury. The MCL repair group had 1.3 mm less absolute medial opening and 0.8 mm less side-to-side difference than the nonoperative treatment group. In a similar prospective comparison of ACL-MCL injuries, Ballmer et al.^23^ found that at 14 months postoperatively, 2 (17%) of 14 MCL injuries (10 grade II, 4 grade III) still had grade I MCL valgus laxity when the MCL was treated nonoperatively. Residual MCL laxity has been shown to be an important risk factor for cruciate ligament graft failure, due to the load-sharing nature of the MCL with the cruciate ligaments during anteromedial rotation and valgus moment.^2^, ^3^, ^4^, ^5^, ^6^ A recent registry study from the Swedish National Knee Ligament Registry included almost 19,000 patients and reported that ACL failure was significantly more likely when the MCL was treated nonoperatively, but not when the MCL was repaired or reconstructed.^6^ Preventing residual valgus laxity is therefore critical to protecting the cruciate ligaments in the MLIK patient.

The strength provided by the additional SA device may explain the valgus stability seen in the current study. SA devices have been the most recent development in ligament repair and have demonstrated similar biomechanical tensile properties to autograft reconstructions, with the added benefit of avoiding donor site morbidity.^25^, ^26^, ^27^, ^28^, ^29^, ^30^ Using a similar repair technique to our own, a recent biomechanical study compared ACL reconstruction combined with either MCL repair or MCL repair with SA. Rotation under valgus load was significantly less after MCL repair with SA as compared with MCL repair alone, suggesting that SA may protect against the forces that cause both MCL and ACL failure.^30^ Clinical evidence of MCL repair with SA is limited.^28^^,^^29^ Paterson-Bynre et al.^57^ reported on a patient treated with open repair with SA for all affected ligaments following a severe KD-IV injury. The patient experienced significant postoperative arthrofibrosis requiring arthroscopic lysis of adhesions at 11 weeks after surgery, with return to sports at 22 weeks, and remained ligamentously stable and active at 5-year follow-up.^57^ The present study is the largest investigation into MCL repair with SA to date and suggests that SA in MCL repair provides reliable valgus stability.

### Limitations

There are limitations to this study. First and most important, valgus laxity outcomes were solely based upon the clinical examination of the senior author, rather than by stress radiograph testing or other objective measurements. Second, baseline PROMs were not available for comparison. Third, no control group was available for comparison, which limits this study to a retrospective case series. Finally, injury patterns and severity of damage to the posteromedial corner was varied, as is the case with most investigations of MLIKs, but this could have affected the outcomes in this study.

## Conclusions

In this study, 20 heterogenous patients with MLIKs treated with acute percutaneous sMCL repair with SA had excellent stability, low rates of postoperative stiffness, and good-to- excellent PROMs at short-term follow-up.

## Supplementary Data

ICMJE author disclosure forms
